# Leucine-rich diet alters the eukaryotic translation initiation factors expression in skeletal muscle of tumour-bearing rats

**DOI:** 10.1186/1471-2407-7-42

**Published:** 2007-03-06

**Authors:** Gislaine Ventrucci, Maria Alice R Mello, Maria Cristina C Gomes-Marcondes

**Affiliations:** 1 Laboratório de Nutrição e Câncer, Departamento de Fisiologia e Biofísica, Instituto de Biologia, Universidade Estadual de Campinas (UNICAMP), Campinas, 13083-970, São Paulo, Brazil; 2 Departamento de Fisiologia e Biofísica, Instituto Biociências, Universidade Estadual de São Paulo, UNESP, Rio Claro, 13506-900, São Paulo, Brazil

## Abstract

**Background:**

Cancer-cachexia induces a variety of metabolic disorders on protein turnorver, decreasing protein synthesis and increasing protein degradation. Controversly, insulin, other hormones, and branched-chain amino acids, especially leucine, stimulate protein synthesis and modulate the activity of translation initiation factors involved in protein synthesis. Since the tumour effects are more pronounced when associated with pregnancy, ehancing muscle-wasting proteolysis, in this study, the influence of a leucine-rich diet on the protein synthesis caused by cancer were investigated.

**Methods:**

Pregnant rats with or without Walker 256 tumour were distributed into six groups. During 20 days of experiment, three groups were fed with a control diet: C – pregnant control, W – tumour-bearing, and P – pair-fed, which received the same amount of food as ingested by the W group; three other groups of pregnant rats were fed a leucine-rich diet: L – pregnant leucine, WL – tumour-bearing, and PL – pair-fed, which received the same amount of food as ingested by the WL group.

**Results:**

The gastrocnemius muscle of WL rats showed increased incorporation of leucine in protein compared to W rats; the leucine-rich diet also prevented the decrease in plasma insulin normally seen in W. The expression of translation initiation factors increased when tumour-bearing rats fed leucine-rich diet, with increase of ~35% for eIF2α and eIF5, ~17% for eIF4E and 20% for eIF4G; the expression of protein kinase S6K1 and protein kinase C was also highly enhanced.

**Conclusion:**

The results suggest that a leucine-rich diet increased the protein synthesis in skeletal muscle in tumour-bearing rats possibly through the activation of eIF factors and/or the S6kinase pathway.

## Background

Cancer-cachexia induces a variety of metabolic disorders, including marked weight loss, especially in adipose and muscle tissues [1]. Skeletal muscle loss causes alterations in protein metabolism that involve a decrease in protein synthesis and an increase in protein degradation [2,3]. Insulin, other hormones, and nutrients such as branched-chain amino acids [4], stimulate protein synthesis.

Leucine, a branched-chain amino acid, stimulates muscle protein synthesis and modules the activity of various proteins involved in the control of mRNA translation [5,6]. Leucine can stimulate protein synthesis directly and/or via its metabolite, α-ketoisocaproic acid (KIC) [7]. Protein synthesis is regulated by interactions between mRNA, tRNA and eukaryotic initiation factors (eIFs). Leucine can stimulate translation via the mammalian target of rapamycin (mTOR) or independently [8-10]. In addition, leucine can stimulate glucose uptake by protein kinase C (PKC), in contrast to insulin which modulates glucose uptake via protein kinase B (PKB) [11].

During the early stages of rat pregnancy, protein is synthesized and accumulated in the maternal organism and is later mobilized during the last days of pregnancy to provide substrates for the fetus [12]. To some extent, the fetus can be compared to rapid tumor growth since both tissues have an exponential growth and are dependent on an adequate supply of glucose and amino acids [13]. For this reason, the association between cancer and pregnancy may change the nutritional supply to both tumour and fetal tissues. Our previous results showed that the total body protein wasting can be enhanced in this situation [13,14] and prevented by nutritional supplementation with leucine [14]. Knowing this, the aim of this study was to examine the effects of a leucine-rich diet on the increased protein degradation and reduced protein synthesis seen in the skeletal muscle of tumour-bearing pregnant rats.

## Methods

### Animals and diets

Young female Wistar rats (45 days old, n = 60) were obtained from the animal facilities of the State University of Campinas (UNICAMP). The rats were housed overnight with adult males (4 females: 1 males), using the harem method [15]. The first day of pregnancy was determined based on the detection of sperm in the vaginal smear. All pregnant rats were then housed in collective cages with free access to water and food under standard conditions (22 ± 2°C, 12/12-h light/dark cycle). During 20 days, the animals were fed with the semipurified diets which consisted of a balanced control diet (C, 18% protein, AIN-G93) and a leucine-rich diet (L, 15% protein with 3% leucine), both containing the same amount of carbohydrate (63%), fat (7%) and fibres (5%), in accordance with the AIN-G93 recommendations [16]. Corn Products Brazil Ingredients supplied some of the diet components, (corn standard and dextrin), whereas the vitamin mix was from DSM Nutritional Products Brazil Ltda., and the amino acids were from Ajinomoto Interamericana Ind. & Com. Ltda., Brazil. The pregnant rats were randomly distributed into six groups. Three groups were fed the control diet: C – pregnant control, W – tumour-bearing, and P – pair-fed, with the latter receiving the same amount of food as ingested by the W group; three other pregnant groups were fed the leucine-rich diet: L – pregnant leucine, WL – tumour-bearing, and PL – pair-fed, the latter receiving the same amount of food as ingested by the WL group.

### Tumour implantation

The rats assigned to the tumour-bearing groups, W and WL, received a subcutaneous injection of Walker 256 tumour cells (approx. 0.25 × 10^6 ^viable cells in 0.5 mL of saline solution) in the right flank after the confirmation of pregnancy. As a standard procedure, the pregnant groups without tumours (C, L, P and PL) received a single injection of 0.5 mL of 0.9% NaCl (w/v) in the right flank. All groups were monitored for 20 days after tumour implantation. The general UKCCCR [17] guidelines for animal welfare were followed, and the protocols were approved by the institutional Committee for Ethics in Animal Research (CEEA/IB/UNICAMP, protocol 217-5).

### Experimental procedures

The rats were killed 20 days after tumor implantation and the gastrocnemius muscles were collected to analyse the incorporation of leucine into muscle protein, and to assess the expression of eukaryotic initiation factors, S6K1 and PKC. Plasma samples were collected to measure the insulin concentration by radioimmunoassay (RIA) [18]. Leucine incorporation was assayed in right gastrocnemius muscles, which were weighed and placed in Krebs-Henseleit bicarbonate (KHB) buffer (composition, in mM: NaCl 110, NaHCO_3 _25, KCl 3.4, CaCl_2 _1, MgSO_4 _1, KH_2_PO_4 _1, glucose 5.5, pH 7.4) supplemented with 1.26 mmol/L leucine. The muscles were pre-incubated separately for 30 min at 37°C with continuous aeration (95% O_2_-5% CO_2_) and shaking, as described by Vary *et al*. [19]. After this period, new KHB buffer supplemented with [^3^H]-leucine (10 μCi.L^-1^; Amersham Biosciences, Little Chalfont, Buckinghamshire, UK) was added and the incubation was continued for a further 2 h. At the end of this period, the muscles were homogenised in trichloroacetic acid (TCA, 1:3 w/v), centrifuged at 10,000 × *g *for 15 min at 4°C and the pellet was suspended in 1 M NaOH and incubated at 40°C for 30 min. Aliquots of this supernatant were used to measure the total protein [20] and to quantity the radioactivity by liquid scintillation using a β-counter. The rates of incorporation were calculated based on the amount of radioactive leucine incorporated in a 2 h period and expressed as pmols [^3^H]-Leu per mg of muscle protein [19].

### Quantification of eIF2, eIF4E, eIF4G, eIF5, p70 S6K1 and PKC in gastrocnemius skeletal muscle

Aliquots of muscle samples were weighed and homogenised in buffer (20 mM N-2-hydroxyethylpiperazine-N-2-ethanesulfonic acid, 100 mM KCl, 0.2 mM EDTA, 2 mM EGTA, 1 mM dithiothreitol, 50 mM NaF, 1 mM 3,3'-diaminobenzide tetrahydrochloride (DAB), 0.5 mM orthovanadate, and 50 mM glycine, pH 7.4) followed by centrifugation at 10,000 × *g *for 15 min at 4°C. Skeletal muscle proteins were resolved by SDS-PAGE on 12% gels followed by transfer of the proteins to a 0.45 μm Hybond-C nitrocellulose membrane (Amersham). All of the antibodies were purchased from Santa Cruz Technology (Santa Cruz, CA, USA). The level of eIF2α, eIF4E and eIF4G expression was assessed using a monoclonal rat antibody against eIF2α, eIF4E and eIF4G (diluted 1:750) followed by detection with a secondary anti-goat horseradish peroxidase (HRP)-labelled antibody (diluted 1:1000). The expression of eIF5 was assessed using an anti-rabbit monoclonal eIF5 (1:750) followed by detection with a secondary anti-rabbit antibody (1:1000). p70S6 kinase expression was assessed using a specific anti-mouse monoclonal S6K1 (1:1000) followed by detection with a secondary anti-mouse antibody (1:1000). Protein kinase C was detected with an anti-mouse antibody against PKC (1:1000) and a secondary monoclonal anti-mouse antibody HRP. Actin (Boehringer Mannheim, Germany) was used as the loading control and was probed with a mouse anti-actin antibody. All of the blots were developed using enhanced chemiluminescence (ECL) reagent (Amersham) and the immunoreactive bands were quantified by densitometry. Images of the gels were captured (FTI 500 Image Master VDS, Pharmacia Biotech) and densitometric analyses of the bands were done using Gel Pro Analyser software (Media Cybernetics, Silver Spring, MD, USA).

### Statistical analysis

The results were expressed as the mean ± SEM. The statistical comparisons were done using one-way ANOVA [21] followed by Bonferroni's test for comparisons among groups. All statistical calculations were done using GraphPad Prism software, v3.00 (GraphPad Inc., San Diego, CA, USA). A value of P < 0.05 indicated significance.

## Results

**Figure 1:**
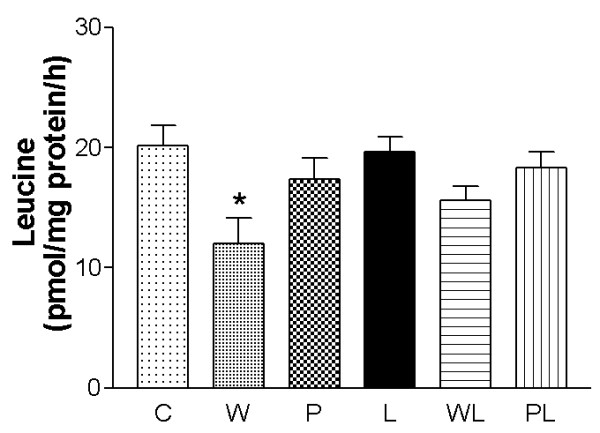
Effects of a leucine-rich diet on leucine incorporation into protein in gastrocnemius skeletal muscle from pregnant, tumour-bearing rats. Right gastrocnemius muscles from all experimental groups were incubated with [^3^H]-L-Leucine for 2 h. For details, see Methods. At least 10 rats were used per group. Abbreviations: C, control; W, tumour-bearing rats; P, pair-fed tumour-bearing rats; L, leucine-rich diet group; WL, tumour-bearing rats fed a leucine-rich diet; PL, pair-fed tumour-bearing group fed a leucine-rich diet. The columns are means ± SEM. * P < 0.05 estatistically different compared to all groups.

The incorporation of leucine into gastrocnemius muscle protein is shown in Figure 1. Tumour growth reduced leucine incorporation in the tumour-bearing groups, W and WL, compared to the respective control groups, C and L. However, the leucine incorporation in the tumour-bearing group fed the leucine-rich diet (WL) was not significantly different from that of the L group. The incorporation of leucine into muscle protein was ~23% lower in the tumour-bearing group (W = 12.00 ± 2.17 pmol [^3^H]-Leu/mg/h) compared to the WL group (15.61 ± 1.21 pmol [^3^H]-Leu/mg/h). The pair-fed groups, P and PL, showed no significant difference compared to the C and L groups (Figure 1). Recent studies have shown that skeletal muscle protein synthesis is stimulated by branched-chain amino acids, especially leucine, administered orally or in perfused muscle [22-24].

Leucine and other branched-chain amino acids stimulate insulin synthesis and release in rats [25]. Tumour growth markedly decreased the plasma insulin concentration in the group W compared to groups C and P (C = 6.013 ± 1.000 ng.mL^-1^; W = 0.785 ± 0.077 ng.mL^-1^*; P = 3.500 ± 0.408 ng.mL^-1^;*p < 0.05 compared to C and P).This finding agreed with other studies in Walker tumour-bearing rats that reported a reduction in the plasma insulin level caused by a decrease in insulin synthesis and release by the β-cells of pancreatic islets [26,27]. The leucine-rich diet prevented the decrease in plasma insulin level associated with tumour growth in the WL group (L = 6.216 ± 0.872 ng.mL^-1^; WL = 3.547 ± 0.286*; PL = 5.019 ± 0.552; *p < 0.05 compared to C and P). In addition, the decrease in plasma insulin concentration in the WL group (~1.7-fold)was significantly less than in the W group (~7.6-fold).

**Figure 2:**
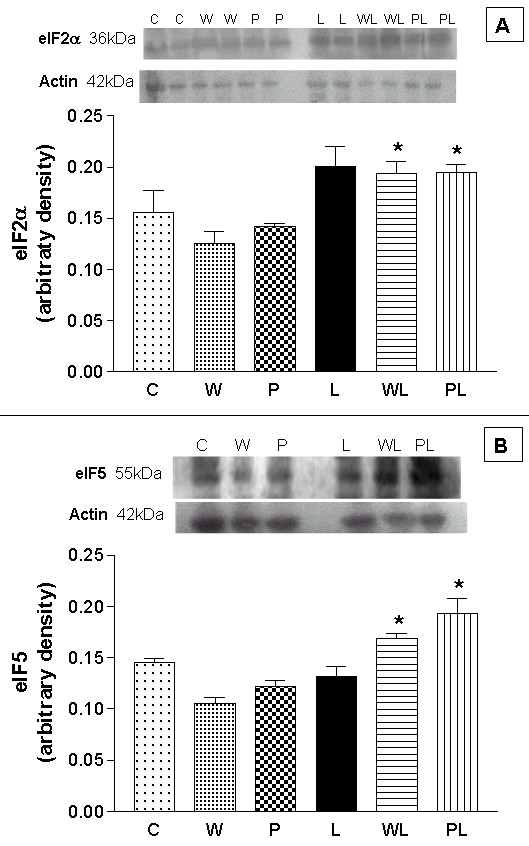
Effects of a leucine-rich diet on the expression of initiation factors in gastrocnemius skeletal muscle from pregnant, tumour-bearing rats. Western blot images and arbitrary densitometric values for the expression of eIF2α (A) and eIF5 (B). Aliquots of gastrocnemius muscle homogenates were loaded onto SDS-PAGE gels (5 μg protein/well) and, after electrophoresis, the proteins were transferred to nylon membranes and blotted with antibodies to eIF2α and eIF5 (1:750 dilution). For details, see Methods. Abbreviations are defined in the legend to Figure 1. The columns are the means ± S.E.M. of at least 10 rats per group. There was no variation in the expression of actin (42 kDa). * P < 0.05, for comparison WL *vs *W and PL *vs *P.

Protein synthesis is a complex process that involves a number of protein factors and ribosomal structures. The initiation of protein synthesis requires eIF2 activity that induces the GTP-dependent binding of Met-tRNAi to the 40S ribosomal subunit to form a preinitiation complex [3,28]. The expression of eIF2α (Figure 2A) increased in the groups fed a leucine-rich diet. The increase in eIF2α expression was ~22% in the L group, but was not significantly different from the control group C. In groups WL (0.194 ± 0.012 arbitrary densitometric units) and PL (0.195 ± 0.007 arbitrary densitometric units), the elF2α expression increased by ~35% and ~27% compared to groups W (0.126 ± 0.012) and P (0.142 ± 0.003), respectively. Factor eIF5 catalyses the binding of GTP to eIF2 factor in the preinitiation complex [29]. In our experiments, eIF5 expression was higher in WL than in W rats (0.1685 ± 0.0052 *vs*. 0.1054 ± 0.0056 arbitrary densitometric units; p < 0.001) (Figure 2B).

**Figure 3:**
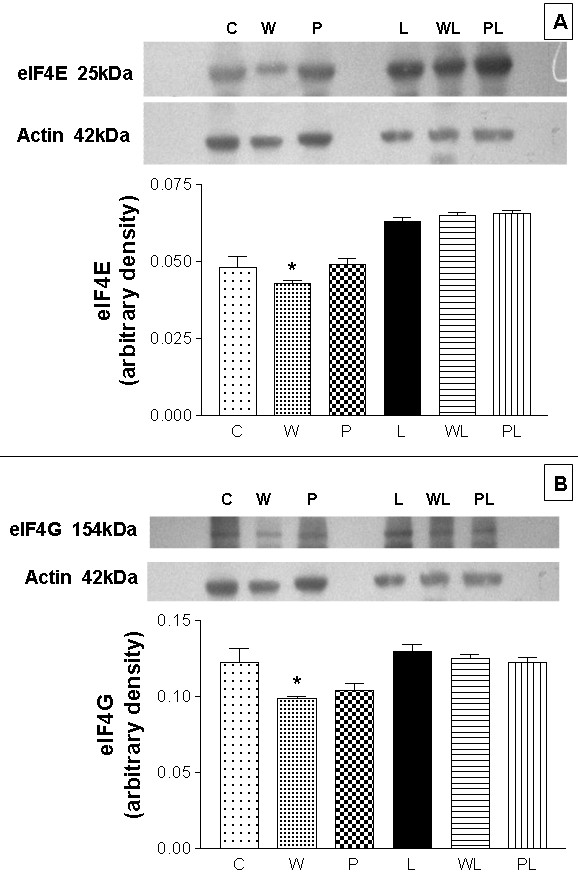
Effects of a leucine-rich diet on the expression of initiation factors in gastrocnemius skeletal muscle from pregnant, tumour-bearing rats. Western blot images and arbitrary densitometric values for the expression of eIF4E (A) and eIF4G (B). Aliquots of gastrocnemius muscle homogenates were loaded onto SDS-PAGE gels (5 μg protein/well) and, after electrophoresis, the proteins were transferred to nylon membranes and blotted with antibodies to eIF4G and eIF4E (1:750 dilution). For details, see Methods. Abbreviations are defined in the legend to Figure 1. The columns are the means ± S.E.M. of at least 10 rats per group. There was no variation in the expression of actin (42 kDa). * P < 0.05 for comparison with all groups.

The activity of the eIF4F complex, which involves the assembly of three initiation factors, including eIF4E and eIF4G, is necessary for recognition and binding of the 43S complex to the 5' terminal of mRNA and for optimal translation [29,30]. Expression of the subunit eIF4E, which recognises the 5' terminal m7GTP cap structure at the 5' end of eukaryotic mRNA [30], was significantly reduced in W rats compared to the other groups, especially WL rats (Figure 3A; C = 0.0516 ± 0.0009, W = 0.0429 ± 0.0014*, P = 0.0468 ± 0.0023, L= 0.0485 ± 0.0008, WL = 0.0519 ± 0.0013 and PL = 0.0528 ± 0.0014 arbitrary densitometric units; *p < 0.01 compared to groups C, WL and PL). Similarly, eIF4G, the subunit that binds to the 43S preinitiation complex, was less expressed in tumour-bearing rats (W = 0.0987 ± 0.0014 arbitrary densitometric units) compared to the other groups (C = 0.1222 ± 0.0093, P = 0.1040 ± 0.0045, L = 0.1329 ± 0.0066 and PL= 0.1101 ± 0.0037 arbitrary densitometric units), while the leucine-rich diet significantly increased eIF4G expression by ~20% in the WL group compared to W (0.1253 ± 0.0024 *vs*. 0.0987 ± 0.0014 arbitrary densitometric units, p < 0.05) (Figure 3B).

**Figure 4:**
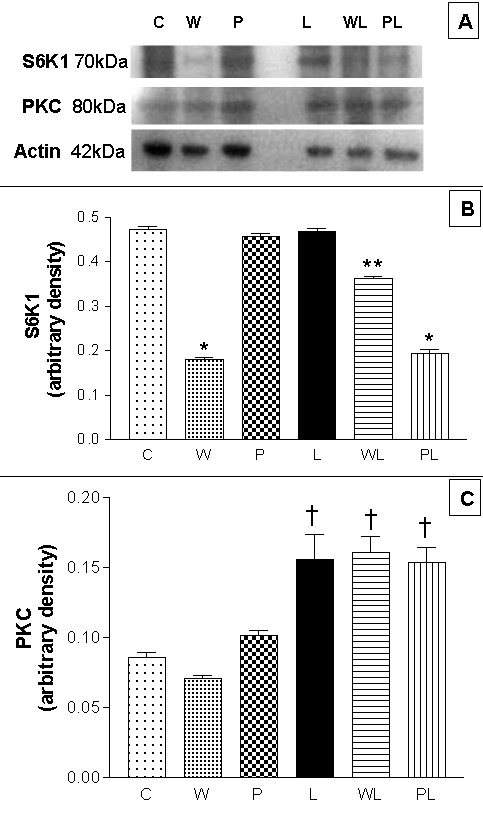
Effects of a leucine-rich diet on the expression of initiation factors in gastrocnemius skeletal muscle from pregnant, tumour-bearing rats. (A) Western blot images of S6K1 (70 kDa), PKC (80 kDa) and actin (42 kDa). These images are representative of the best assay from each group (minimum of 10 rats per group). (B) Arbitrary densitometric values for S6K1 expression from western blots of eight rats per group. (C) Arbitrary densitometric values for PKC expression from western blots of eight rats per group. Aliquots of gastrocnemius muscle homogenates were loaded onto SDS-PAGE gels (5 μg protein/well) and, after electrophoresis, the proteins were transferred to nylon membranes and blotted with antibodies to eIF2α and eIF5 (1:1500 dilution). For details, see Methods. Abbreviations are defined in the legend to Figure 1. The columns are the means ± S.E.M. of at least 8 rats per group. There was no variation in the expression of actin (42 kDa). * P < 0.05 for comparison W *vs *C and WL, PL *vs *P; ** P < 0.05 for comparison WL *vs *L; † P < 0.05 for comparison L *vs *C, WL *vs *W, PL *vs *P.

The oral administration of leucine increases the phosphorylation of S6K1 in rat muscles [31]. There was a reduction in S6K1 expression in the gastrocnemius muscle of W rats compared to C rats (0.182 ± 0.005 *vs*. 0.473 ± 0.007 arbitrary densitometric units) (Figure 4A (WB image) and 4B). Although the expression of S6K1 in WL rats was lower than in L rats (0.362 ± 0.005 *vs*. 0.470 ± 0.005 arbitrary densitometric units), the expression of this kinase was ~32% higher in WL rat muscle than in W rats (p < 0.001). PKC expression was significantly higher (p < 0.001) in all groups fed the leucine-rich diet (L = 0.156 ± 0.018, WL = 0.161 ± 0.012, PL = 0.154 ± 0.011 arbitrary densitometric units) compared to those fed the control diet (C = 0.086 ± 0.003, W = 0.071 ± 0.002 and P = 0.102 ± 0.003 arbitrary densitometric units). However, there was no significant difference between the WL and L (control) groups (0.161 ± 0.011 *vs*. 0.156 ± 0.018 arbitrary densitometric units) (Figure 4A (WB image) and 4C), although this parameter was 2.27-fold higher in WL than in W rats.

## Discussion

Cachexia, a severe condition in cancer patients, involves wasting of the body tissues with a loss of muscle mass that may reduce performance, the response to chemotherapy, and life expectancy. Since the depletion of lean body mass in cancer cachexia is the major factor in decreasing the survival time, an improvement in protein synthesis and/or a reduction in protein catabolism could interfere with physiological processes involved in patient welfare. As shown here, a leucine-rich diet improved the protein metabolism of muscle tissue by enhancing the expression of eukaryotic initiation factors that is normally depressed during tumour growth. Since protein synthesis is reduced and protein degradation is increased throughout the body during cancer cachexia, the incorporation of amino acids, especially branched-chain amino acids, by muscle cells could supply important nutrients and influence protein synthesis. The lower incorporation of leucine into gastrocnemius muscle proteins in tumour-bearing rats indicated a decrease in protein synthesis that paralleled an increase in protein wasting, as previously reported by ourselves [1,2,32] and others [33,34]. As shown here, the leucine-rich diet reduced the protein catabolism and increased the protein synthesis in WL rats compared to W rats.

The rate of protein synthesis depends on the nutritional and physiological status of the animal [35-37], including the availability of dietary protein and amino acids [8,38], although in the pair-fed groups studied here (P and PL) there was no reduction in leucine incorporation into muscle protein compared to the tumour-bearing groups. In animals and human pregnancy [12-14,36,37] the protein synthesis is highly activated, especially during the early stages of pregnancy. The findings in pair-fed groups suggested that food restriction caused less wasting of muscle protein than did tumour growth. Even at the late stage of pregnancy in rat, there is protein synthesis associated to high proteolysis [12-14,36,37], providing substracts to fetus and also to tumor cells [14]. This fact suggests that the rapid tumor growth-induced hypercatabolism in pregnant rats shows more evident effects on protein turnover.

In recent years, the impact of nutrients on cell signalling events and the regulation of protein synthesis by branched-chain amino acids have become an important area of research [39]. Protein synthesis involves many eukaryotic initiation factors (eIFs) that are involved in a complex, multistage process regulated by three steps [29,40]. The first step involves association of the 40S ribosomal subunit with eIF2 and GTP to form a ternary complex (eIF2-GTP-Met-tRNA), with eIF2α being a key target in translational regulation [41]. The reduction in skeletal muscle protein synthesis seen during cancer cachexia (W group, previously observed in our study [2]) was associated with a decrease in eIF2α and eIF5 expression and a reduction in leucine incorporation, indicating the ability of tumour growth to produce important tissue wasting. On the other hand, eIF2α expression was strongly enhanced in the leucine-treated groups (L, PL and especially LW) and was correlated with increased eIF5 expression and the maintenance of leucine incorporation. Since eIF5 is a catalytic factor for many translation factors, including the release of eIF2-GDP from met-tRNA, then once the eIF2-GDP complex is released it may participate in another round of initiation, with GDP being exchanged for GTP prior to the formation of a new eIF2-GTP-met-t RNA complex.

The second step in the initiation of translation involves the binding of mRNA to the 40S ribosomal subunit, an interaction that is mediated by a triad of initiation factors including eIF4E, which associates with eIF4G to form an active complex (eIF4F) [29,35,39,40]. The decreased eIF4E expression seen in tumour-bearing rats suggested that there was less translation and, consequently, less protein synthesis since eIF4E is necessary for assembly of the eIF4G-eIF4E complex which modulates the overall rate of protein synthesis. The enhanced formation of eIF4F may involve the phosphorylation of eIF4G, which is correlated with the stimulation of protein synthesis [42]. Similarly, decreased eIF4G-eIF4E assembly and a corresponding decrease in eIF4G phosphorylation in the presence of mTOR activation has been observed in the skeletal muscle of septic rats [43,44].

The leucine-dependent stimulation of mRNA translation is partly insensitive to rapamycin [45]. This finding is supported by the observation that skeletal muscle protein synthesis and eIF4E-eIF4G assembly are enhanced by the administration of leucine in the presence of rapamycin (a specific inhibitor of mTOR) [45]. Additionally, oral leucine administration enhanced the phosphorylation of 4E-BP1, showing the stimulation effect of leucine on protein synthesis in skeletal muscle of normal rats [46]. Conversely, leucine alone has beneficial effects on skeletal muscle metabolism in tumour-bearing animals [2]. As shown here, leucine tended to increase the expression of eIF4G in skeletal muscle. This slight increase in the amount of eIF4G could result in greater binding to eIF4E. The regulation of the initiation of translation depends on the availability of eIF4E [35], the levels of which were maintained in the leucine-rich diet group (LW). The oral administration of isoleucine, which is less effective than leucine, activates the initiation of translation in skeletal muscle in rats [23], and the binding of eIF4G to eIF4E is enhanced by isoleucine and leucine in rats [27,31,47]. Another mechanism for regulating protein synthesis is the phosphorylation of the ribosomal protein S6 that is mediated by the 70 kDa ribosomal protein S6 kinase (S6K1) [48]. The activation of S6K1 increases the capacity to synthesise protein [39,45].

The cellular mechanisms by which amino acids modulate protein synthesis are now beginning to be understood. Amino acids, and leucine in particular, consistently activate S6K1 and promote translation [49]. Among branched-chain amino acids, leucine apparently has the greatest effect in activating S6K1 [50,51]. Since increased S6K1 activity has been associated with increased protein synthesis and enhanced translation of mRNA [52], our results suggest that the leucine pathways in muscle protein synthesis require an increased availability of eIF4E, and S6K1 activity. The availability of leucine could activate S6K1, as verified by Yoshizawa and collegues [46,53] thereby enhancing the protein synthesis in gastrocnemius muscle, as seen in the WL, L and PL groups. Although Yoshizawa and collegues [46,53] observed increase on translational factos activity after 1 hour leucine feeding, the authors found that this stimule reduced to basal level after 3 h. Controvertly, in the present study, probably the increased on eIF4E, and S6K1 were directely related to the constant stimule by the leucine-rich diet intake, during 20 days. This activation could be mediated by mTOR through different mechanisms [54].

Several reports have suggested a possible interplay between leucine, mitochondrial function and mTOR signalling [55,56]. Xu *et al*. [57] proposed that leucine stimulates S6K1 phosphorylation via the mTOR pathway, partly by serving as a mitochondrial fuel through oxidative carboxylation and partly by mediating the allosteric activation of glutamate dehydrogenase, thereby decreasing the activity of AMP-activated protein kinase (AMPK). Leucine enhances glucose uptake by the PKC-dependent pathway [4,11], and the increased PKC expression in all of the groups fed a leucine-rich diet suggested that leucine improved the glucose uptake and minimised the effects of cancer-cachexia in skeletal muscle (WL).

When amino acids and glucose are present in adequate amounts, AMPK, which usually inhibits the mTOR-S6K1 pathway, is depressed and there is an increase in protein synthesis [55]. Although cancer-cachexia decreases plasma insulin levels [58,59], the leucine-rich diet improved the plasma insulin level in the LW group, and could serve as a stimulus for protein synthesis [60]. The oral administration of leucine in vivo results in transient increases in circulating insulin, but blocking this response with somatostatin prevents the leucine-induced hyperphosphorylation of 4E-BP1 and S6K1 in rat skeletal muscle, whereas formation of the eIF4E-eIF4G complex is increased [25,31]. However, amino acids do not stimulate phosphatidylinositol 3-kinase(PI3-K) or protein kinase B (PKB) (Akt) signalling pathways activated by insulin and growth factors [50,61,62]. Bolster and colleagues [63] reported that leucine had no effect on 4E-BP1 and S6K1 during skeletal muscle protein synthesis, in contrast to the findings of studies in vivo [23] and in vitro [64] which suggested that the residual insulin signalling seen in postabsorptive state rats provided the basal phosphorylation for 4E-BP1 and S6K1; however, this level of signalling was insufficient to cause hyperphosphorylation, regardless of the availability of leucine.

Although the combined effect of leucine and insulin provides an integrated response, leucine alone may preferentially enhance the expression and activity of selected components of translation initiation, thereby increasing protein synthesis. As shown here and elsewhere [2], a leucine-rich diet increased protein synthesis in skeletal muscle of tumour-bearing rats and thereby enhanced the levels of many signalling factors. The finding that leucine influenced the synthesis of eIF factors and/or activated the S6kinase pathways could lead to new alternatives for complimentary treatment during cancer therapy. Specifically, we are currently investigating whether leucine supplementation can counteract body weight loss during cancer cachexia by inducing protein synthesis.

## Conclusion

The oral administration of leucine-rich diet induced the increase on the protein synthesis in skeletal muscle in tumour-bearing rats possibly through the activation of eIF factors and/or the S6kinase pathway.

## Competing interests

The author(s) declare that they have no competing interests.

## Authors' contributions

The author G.V. contributed to the data collection, analysis, data interpretation, and manuscript preparation. The author M.A.R.M. participated in the design of the study and contributed to manuscript preparation. The author M.C.C.G.M. conceived the study and its design and coordinated the works and manuscript preparation. All authors read and approved the final manuscript.

## Pre-publication history

The pre-publication history for this paper can be accessed here:
